# Prognostic and recurrent significance of SII in patients with pancreatic head cancer undergoing pancreaticoduodenectomy

**DOI:** 10.3389/fonc.2023.1122811

**Published:** 2023-05-22

**Authors:** Qing Chen, Siqian Ren, Songping Cui, Jincan Huang, Di Wang, Binglin Li, Qiang He, Ren Lang

**Affiliations:** ^1^ Department of Hepatobiliary and Pancreaticosplenic Surgery, Beijing ChaoYang Hospital, Capital Medical University, Beijing, China; ^2^ Department of General Surgery, Peking University Third Hospital, Beijing, China

**Keywords:** pancreatic head carcinoma, surgery, systemic immune-inflammation index, prononsis, recurrence

## Abstract

**Background:**

To investigate the clinical significance of preoperative inflammatory status in patients with pancreatic head carcinoma (PHC), we performed a single-center study to assess it.

**Method:**

We studied a total of 164 patients with PHC undergoing PD surgery (with or without allogeneic venous replacement) from January 2018 to April 2022. Systemic immune-inflammation index (SII) was the most important peripheral immune index in predicting the prognosis according to XGBoost analysis. The optimal cutoff value of SII for OS was calculated according to Youden index based on the receiver operating characteristic (ROC) curve and the cohort was divided into Low SII group and High SII group. Demographic, clinical data, laboratory data, follow-up data variables were obtained and compared between the two groups. Kaplan-Meier curves, univariable and multivariable Cox regression models were used to determine the association between preoperative inflammation index, nutritional index and TNM staging system with OS and DFS respectively.

**Results:**

The median follow-up time was 16 months (IQR 23), and 41.4% of recurrences occurred within 1 year. The cutoff value of SII was 563, with a sensitivity of 70.3%, and a specificity of 60.7%. Peripheral immune status was different between the two groups. Patients in High SII group had higher PAR, NLR than those in Low SII group (P <0.01, <0.01, respectively), and lower PNI (P <0.01). Kaplan–Meier analysis showed significantly poorer OS and DFS (P < 0.001, <0.001, respectively) in patients with high SII. By using the multivariable Cox regression model, high SII (HR, 2.056; 95% CI, 1.082–3.905, P=0.028) was significant predictor of OS. Of these 68 high-risk patients who recurrence within one year, patients with widespread metastasis had lower SII and worse prognosis (P <0.01).

**Conclusion:**

High SII was significantly associated with poor prognosis in patients with PHC. However, in patients who recurrence within one year, SII was lower in patients at TNM stage III. Thus, care needs to be taken to differentiate those high-risk patients.

## 1 Introduction

Pancreatic ductal adenocarcinoma (PDAC) is a lethal malignant neoplasm with low 5-year survival rate, and surgical resection represents the only path to cure (1). At present, resectable and borderline resectable pancreatic cancer could be access to surgical care according to the NCCN guidelines (2), but unfortunately most patients are advanced at the time of diagnosis, leading an unsatisfactory surgical prognosis (3). Currently, there is no good way to predict the prognosis of patients before surgery by non-invasive method. In recent years, it has been found that tumor-associated inflammatory factors SII, NLR (4), as well as immune-nutritional indicator PNI and PAR (5), are closely related to the outcome of cancer patients. Therefore, we wanted to explore the association between prognosis and inflammatory or nutritional status in pancreatic cancer. As far as we know few studies had reported the prognostic value of SII in predicting PHC undering PD or PD with allogeneic venous replacement.

## 2 Materials and methods

### 2.1 Patients

**Figure 1:**
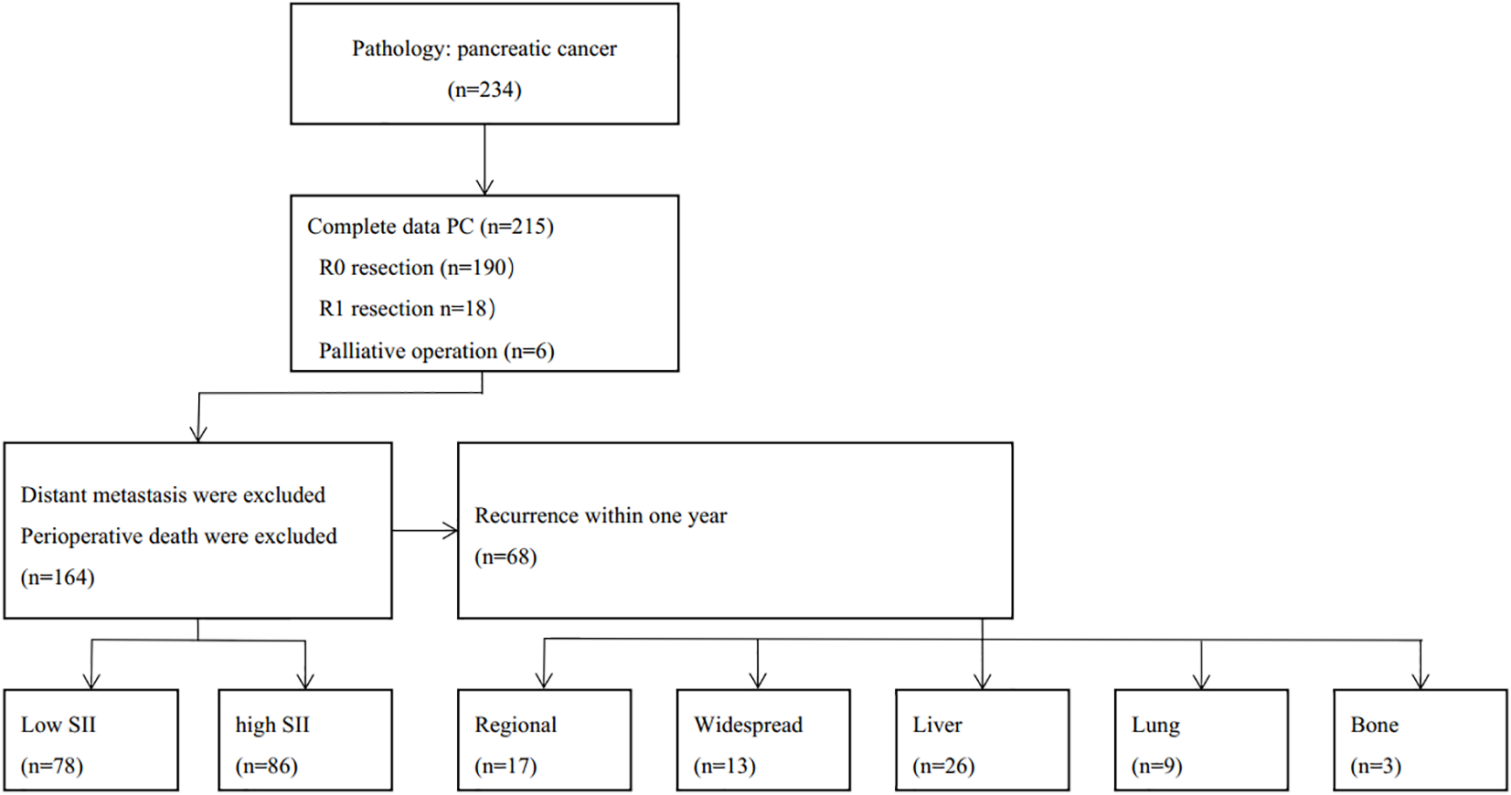
Flow chart of patient’s enrollment in the study.

We retrospectively analyzed the clinical data and follow-up data of patients that met inclusion criteria (**Figure 1**). Inclusion criteria: (1) Patients who were accepted and received surgery in Department of Hepatobiliary Surgery in Beijing Chaoyang Hospital from January 2018 to April 2021; (2) Age between 18 to 80, no limitation on gender; (3) Preoperative imaging confirmed the tumor was located in the head of the pancreas, postoperative pathology was officially diagnosed as pancreatic adenocarcinoma; (4) Received pancreaticoduodenectomy (PD) or PD with allogeneic venous replacement; (5) Patients with tumors suffered no arterial invasion and distal metastasis. Exclusion criteria:(1) Patients with other systemic tumors; (2) R1 resection or palliative operation;(3) Perioperative death; (4) Clinical data and follow-up data were incomplete.

### 2.2 Clinical characteristics and follow-up

The following data were recorded through the electronic medical record system: patient-related information included age, sex, symptom, comorbidities (diabetes, cerebrovascular diseases, etc.); tumor-related information included preoperative imaging examination, tumor diameter, venous invasion, lymph node metastasis, distant metastasis and TNM staging system (The American Joint Commission on Cancer (AJCC) 8th edition staging system for pancreatic ductal adenocarcinoma); Treatment-related information included surgical approach, operation time, blood loss; Routine laboratory tests included white blood cell (WBC), lymphocyte (LYM), platelet (PLT), neutrophil (Neut), direct bilirubin (DB), hemoglobin, albumin, tumor markers (CA19-9, CEA, etc.). The primary outcome of this study was the overall survival and diseases free survival after surgery. Secondary outcome is death from any cause. Complete follow-up data included whether the patient had any recurrence, time to recurrence, location, and time of death. All patients were followed up by telephone, return visit, or inpatient observation, and the follow-up data were recorded until April 2021 or death.

### 2.3 Statistical analysis

**Figure 2:**
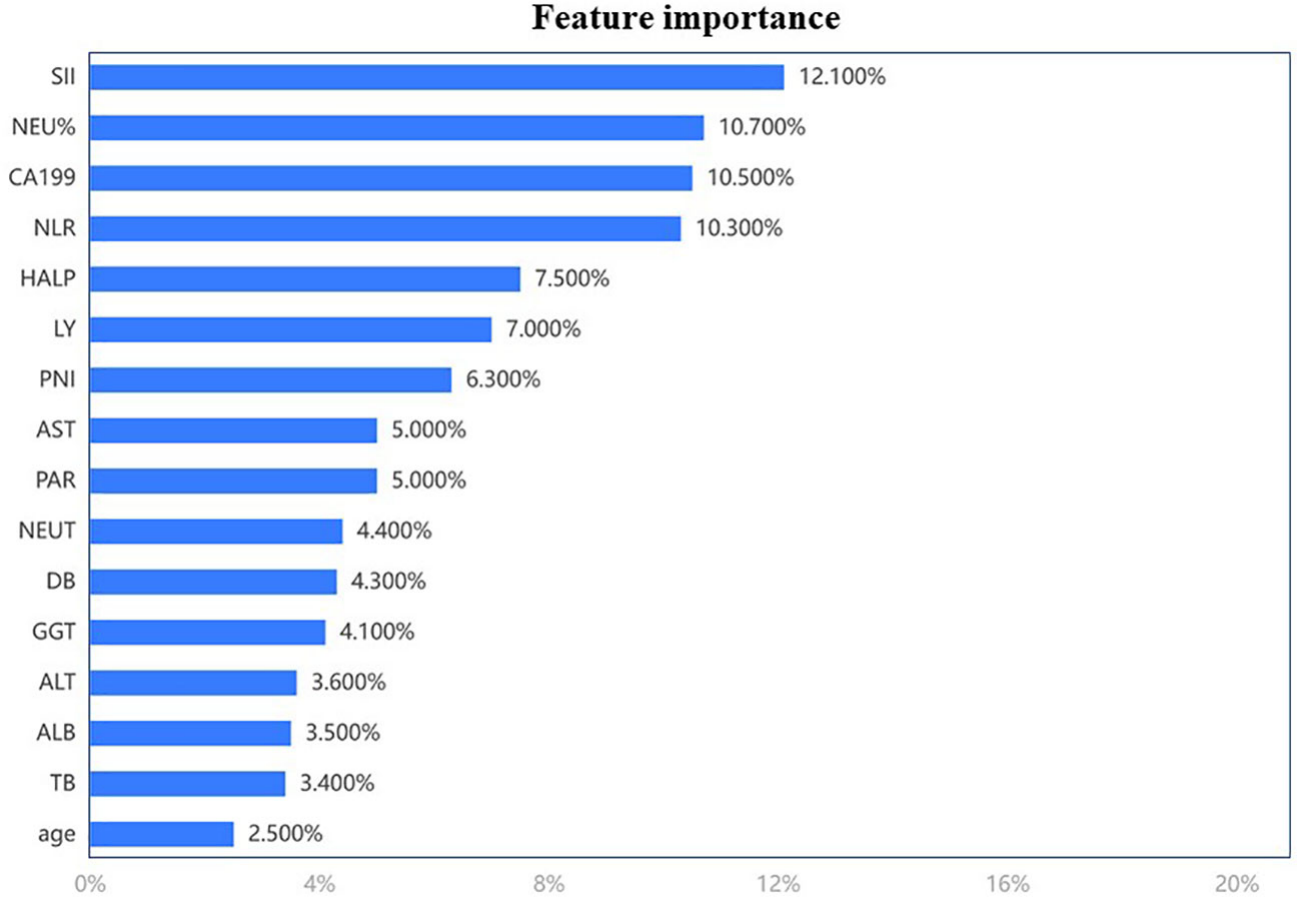
The feature importance associated with survival analyze by eXtreme Gradient Boosting (XGBoost) classifier.

In this study, we used an eXtreme Gradient Boosting (XGBoost) classifier and analyze the feature importance associated with survival (**Figure 2**). XGBoost is a machine learning technique that could assemble weak prediction models to build an accurate one (6). SII was one of the most important peripheral immune factors in predicting the prognosis of this cohort. The optimal cutoff value of SII for OS was calculated according to Youden index based on the receiver operating characteristic (ROC) curve and the cohort was divided into Low SII group and High SII group. Measurement data were presented as mean and standard deviation or median and interquartile range, and count data were presented as numbers (percentages). Measurement data were compared by two unpaired sample t-test, and count data were compared using chi-square test or Fisher’s exact test. Kaplan-Meier curves were applied for survival analysis. Two-sided p<0.05 was considered as statistical significance. All the data were analyzed using SPSS (version 26.0) and R software (version 4.2.0).

## 3 Results

### 3.1 Baseline and clinical characteristics of patients

A total of 164 consecutive patients were recruited in this study, and the the clinical characteristics are shown in **Table 1**. Included 91 males and 73 females with a mean age of 63.8 ± 10.6 years. At the beginning of the research, we used an XGBoost classifier and analyze the feature importance associated with overall survival. SII was the most important preoperative inflammatory and nutritional factors below (**Figure 2**). Patients were divided into 78 (47.6%) patients in the Low SII group and 86 (52.4%) patients in the High SII group by 563 as the cutoff. The demographic and clinical data of the two groups are shown in **Table 1**.There were significant differences in WBC, PLT, LYM, Neut, DB, platelet to albumin ratio (PAR), albumin (g/L)+5×lymphocyte count (109/L) (PNI), and neutrophil/lymphocyte count (NLR) (p <0.01, <0.01, <0.01, <0.01, = 0.01, <0.01, <0.01, <0.01, respectively) between the Low SII group and the High SII group.

**Table 1:** Baseline characteristics of patients with PHC.

Variable	Total	Low-SII (n=78)	High-SII (n=86)	p value
Age, mean (SD), y	63.8 (10.6)	63.6 (10.0)	64.0 (11.2)	0.81
Sex (n/%)				0.09
Male	91 (55.5)	38 (48.7)	53 (61.6)	
Female	73 (44.5)	40 (51.3)	33 (38.4)	
Symptom (n/%)				0.08
Asymptomatic	10 (6.1)	7 (9.0)	3 (3.5)	
Abdominal pain	60 (36.6)	34 (43.6)	26 (30.2)	
Jaundice	89 (54.3)	32 (41.0)	57 (66.3)	
Gastrointestinal symptom	5 (3.0)	5 (6.4)	0 (0.0)	
Cerebrovascular diseases (n/%)	66 (40.2)	31 (39.7)	35 (40.7)	0.92
Smoking (n/%)	48 (29.3)	20 (25.6)	28 (32.6)	0.33
Diabetes (n/%)	45 (27.4)	22 (28.2)	23 (26.7)	0.84
Hepatitis (n/%)	3 (1.8)	2 (2.6)	1 (1.2)	0.51
Biliary drainage	26 (15.9)	11 (14.1)	15 (17.4)	0.56
WBC, mean (SD), 10^9^/L	6.1 (1.9)	5.3 (1.4)	6.9 (2.1)	**<0.01**
Neut, mean (SD), 10^9^/L	3.9 (1.5)	3.1 (1.0)	4.8 (1.4)	**<0.01**
Lym, mean (SD), 10^9^/L	1.5 (0.5)	1.6 (0.6)	1.3 (0.4)	**<0.01**
PLT, mean (SD), 10^9^/L	232.1 (83.2)	190.9 (55.1)	269.5 (86.9)	**<0.01**
HGB, mean (SD), g/L	119.1 (19.1)	121.9 (11.2)	116.5 (21.0)	0.07
ALB, mean (SD), g/L	35.4 (4.9)	36.0 (5.3)	34.8 (4.5)	0.11
AST, mean (SD), U/L	47.5 (96.8)	47.0 (105.5)	52.5 (90.7)	0.98
ALT, mean (SD), U/L	64 (118.3)	60 (120.5)	73.0 (122.3)	0.85
GGT, mean (SD), U/L	288.0 (616.5)	220.5 (651.3)	292.5 (501.5)	0.78
DB, mean (SD), μmol/L	61.5 (124.8)	28.1 (92.1)	73.2 (128.0)	**0.01**
CEA,median (IQR), U/mL	2.5 (3.0)	2.5 (3.1)	2.5 (2.8)	0.47
CA199, median (IQR), U/mL	66.5 (278.5)	48.9 (146.3)	135.4 (881.0)	0.15
PAR, mean (SD)	6.7 (2.6)	5.3 (1.7)	7.1 (2.7)	**<0.01**
PNI, mean (SD)	42.8 (5.9)	43.9 (6.3)	40.7 (5.4)	**<0.01**
NLR, mean (SD)	3.1 (2.3)	2.0 (0.7)	3.6 (2.8)	**<0.01**
OP procedure (n/%)				0.66
PD (n/%)	85 (51.8)	39 (50.0)	46 (53.5)	
PD with allogeneic venous replacement	79 (48.2)	39 (50.0)	40 (46.5)	
Blood loss, median (IQR), mL	500 (400)	500 (400)	550 (400)	0.20
OP time, mean (SD), h	10.1 (2.3)	9.9 (1.9)	10.6 (2.8)	0.99
T-stage (n/%)				0.78
I	31 (18.9)	18 (23.1)	13 (15.1)	
II	46 (28.0)	19 (24.4)	27 (31.4)	
III	8 (5.0)	2 (2.6)	6 (7.0)	
IV	79 (48.1)	39 (50)	40 (46.5)	
N (n/%)				0.83
0	58	28 (35.9)	30 (34.9)	
1	65	29 (37.2)	36 (41.9)	
2	41	21 (26.9)	20 (23.3)	
TNM stage (n/%)				0.72
I	29 (17.7)	15 (19.2)	14 (16.3)	
II	37 (22.6)	17 (21.8)	20 (23.3)	
III	98 (59.7)	46 (59)	52 (60.5)	
IV	0 (0.0)	0 (0.0)	0 (0.0)	
Grading of cancer (n/%)				0.63
Low	47 (28.7)	16 (20.5)	31 (36.0)	
Intermediate	103 (62.8)	56 (71.8)	47 (54.7)	
High	14 (8.5)	6 (7.7)	8 (9.3)	

PHC, pancreatic head carcinoma; SD, standard deviation; WBC, white blood cell; PLT, platelet; Neut, neutrophil; HGB, hemoglobin; ALB, albumin; AST, aspartic transaminase; ALT, alanine aminotransferase; DB, direct bilirubin; GGT, gamma-glutamyl transferase; CEA, carcinoembryonic antigen; CA199, carbohydrate antigen 199; PAR, platelet to albumin ratio; PNI, prognostic Nutritional Index; NLR, neutrophil to lymphocyte ratio; IQR, interquartile range; OP, operation; PD, pancreaticoduodenectomy.

Bold values: P value has statistical significance.

### 3.2 The prognostic value of SII for predicting overall survival

**Figure 3:**
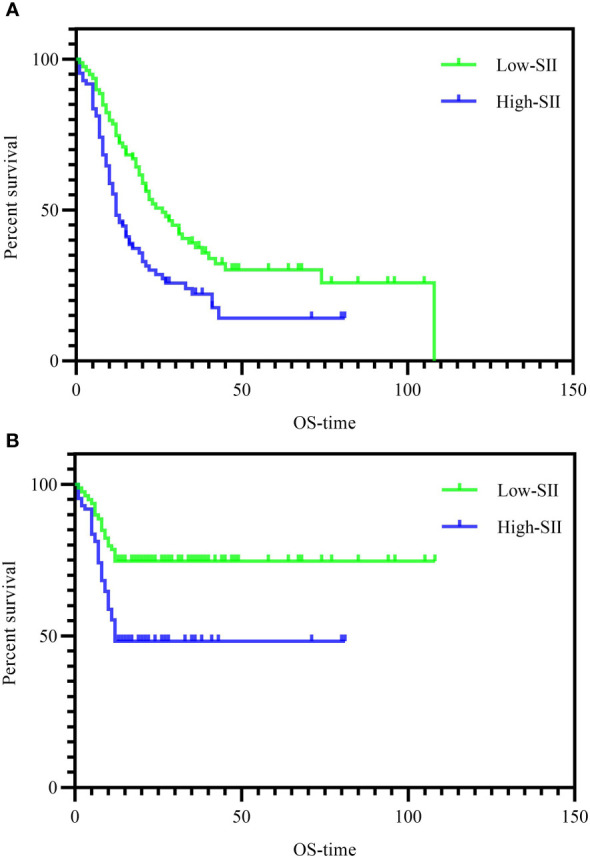
**(A)** Kaplan-Meier curves for OS according to SII level. OS of patients with SII≦563 was significantly longer than that of patients with SII>563 (P=0.001 by log-rank test). OS, overall survival. **(B)** Kaplan-Meier curves for 1-year OS according to SII level (P<0.001 by log-rank test). OS, overall survival.

To further assess the prognostic value of SII in PHC. Univariate analysis and multivariate analysis were performed in **Table 2**. Because there was multicollinearity between peripheral blood mononuclear cells and inflammation index, we only included inflammation index in the regression analysis. TNM staging system as the authoritative prognostic indicator was also included. The results showed that except for PAR, the p values of PNI, NLR, TNM group (PHC was broadly divided into 2 groups according to TNM staging system: the low-risk group: Stage I-II, and the high-risk: Stage III) and SII group were < 0.2. We included these meaningful indicators into the multivariate analysis. The results showed that NLR (HR=1.135; p=0.002), TNM stage III (HR=1.803; p=0.003), High SII (HR=1.386; p=0.009), were independent risk factors of OS. Shorter OS was significantly associated with the higher SII, higher NLR and TNM stage III. The median OS was 22 months and 12 months for patients with SII ≤ 563 and SII > 563, respectively. Further analysis revealed that PNI was a protective factor associated with 1-year survival in patients with PHC (**Table 3**). Kaplan–Meier analysis showed that OS or 1-year OS was significantly shorter in the high SII group than in the low SII group as shown in **Figure 3** (P < 0.001).

**Table 2:** Factors associated with the total OS in the total cohort.

Variable	Univariate analysis		Multivariate analysis	
	HR (95% CI)	p value	HR (95% CI)	p value
PAR	1.039 (0.968-1.115)	0.290		
PNI	0.958 (0.928-0.989)	**0.008**	0.969(0.937-1.001)	0.059
NLR	1.202 (1.126-1.283)	**0.000**	1.135(1.046-1.232)	**0.002**
TNM(S_1~2_ vs S_3_)	1.752 (1.196-2.569)	**0.004**	1.803(1.221-2.662)	**0.003**
SII (Low vs High)	1.837 (1.270-2.656)	**0.001**	1.386(0.930-2.065)	**0.009**

PAR, platelet to albumin ratio; PNI, prognostic Nutritional Index; NLR, neutrophil to lymphocyte ratio; TNM S1~2, TNM stage I ~ TNM stage II ; SII, systemic immune-inflammation index SII = (P × N)/L, where P, N, and L refer to peripheral platelet, neutrophil, and lymphocyte counts, respectively.

Bold values: P value has statistical significance.

**Table 3:** Factors associated with the 1-year OS in the total cohort.

Variable	Univariate analysis		Multivariate analysis	
	HR (95% CI)	p value	HR (95% CI)	p value
PAR	1.073 (0.982-1.172)	0.120	0.970(0.866-1.086)	0.600
PNI	0.935 (0.894-0.978)	**0.003**	0.947(0.901-0.996)	**0.033**
NLR	1.204 (1.125-1.288)	**0.000**	1.110(1.017-1.213)	**0.016**
TNM(S_1~2_ vs S_3_)	2.382 (1.345-3.103)	**0.003**	2.533(1.082-3.905)	**0.002**
SII (Low vs High)	2.572 (1.503-4.400)	**0.001**	2.056(1.082-3.905)	**0.028**

PAR, platelet to albumin ratio; PNI, prognostic Nutritional Index; NLR, neutrophil to lymphocyte ratio; TNM S1~2, TNM stage I ~ TNM stage II ; SII, systemic immune-inflammation index SII = (P × N)/L, where P, N, and L refer to peripheral platelet, neutrophil, and lymphocyte counts, respectively.

Bold values: P value has statistical significance.

### 3.3 The prognostic value of SII for predicting disease free survival

**Figure 4:**
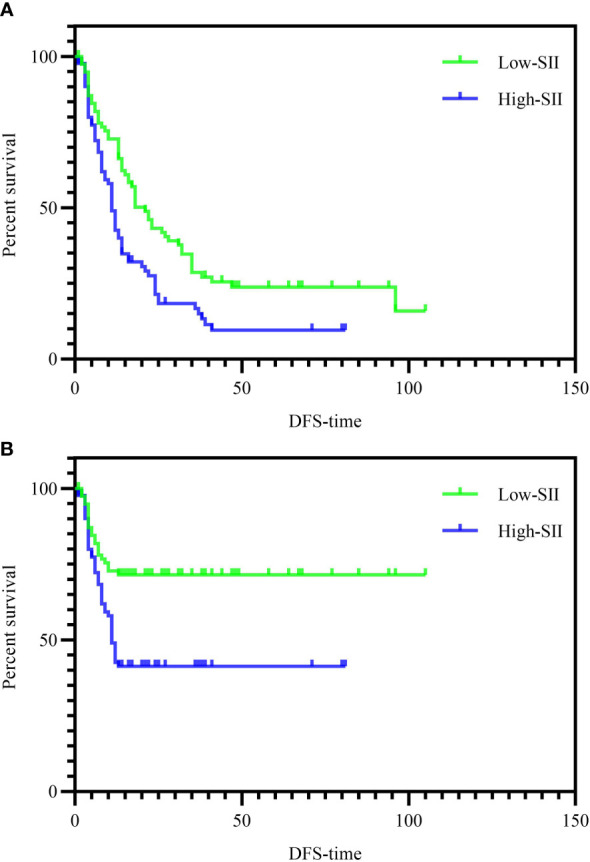
**(A)** Kaplan-Meier curves for DFS according to SII level. DFS of patients with SII≦563 was significantly longer than that of patients with SII>563 (P=0.001 by log-rank test). DFS, disease-free survival. **(B)** Kaplan-Meier curves for 1-year DFS according to SII level (P<0.001 by log-rank test). DFS, disease-free survival.

Univariate analysis was performed in **Table 4**. Among them, the P values of PAR, PNI, NLR, TNM group and SII group were < 0.2. We include these meaningful indicators into the multivariate analysis and the results showed that TNM stage III (HR=2.580; p=0.001), High SII (HR=2.097; p=0.025), were independent risk factors of DFS (**Table 4**). Moreover, PNI (HR =0.945; p = 0.021) was also a factor affecting the DFS. When we set the endpoint event to 1-year DFS, only TNM stage III was an independent risk factor according to multivariate analysis (**Table 5**). Kaplan–Meier analysis showed that DFS or 1-year DFS was significantly shorter in the high SII group than in the low SII group as shown in **Figure 4** (P = 0.001).

**Table 4:** Factors associated with the DFS in the total cohort.

Variable	Univariate analysis		Multivariate analysis	
	HR (95% CI)	p value	HR (95% CI)	p value
PAR	1.028 (0.958-1.102)	0.147	0.971 (0.869-1.084)	0.597
PNI	0.968 (0.939-0.998)	**0.038**	0.945 (0.901-0.991)	**0.021**
NLR	1.213 (1.109-1.327)	**0.000**	1.086 (0.942-1.252)	0.254
TNM (S_1~2_ vs S_3_)	1.657 (1.148-2.393)	**0.007**	2.580 (1.496-4.450)	**0.001**
SII (Low vs High)	1.779 (1.241-2.550)	**0.002**	2.097 (1.100-3.998)	**0.025**

PAR, platelet to albumin ratio; PNI, prognostic Nutritional Index; NLR, neutrophil to lymphocyte ratio; TNM S1~2, TNM stage I ~ TNM stage II ; SII, systemic immune-inflammation index SII = (P × N)/L, where P, N, and L refer to peripheral platelet, neutrophil, and lymphocyte counts, respectively.

Bold values: P value has statistical significance.

**Table 5:** Factors associated with the 1-year DFS in the total cohort.

Variable	Univariate analysis		Multivariate analysis	
	HR (95% CI)	p value	HR (95% CI)	p value
PAR	1.079 (0.990-1.176)	0.083	0.960 (0.876-1.051)	0.376
PNI	0.938 (0.898-0.979)	**0.003**	0.970 (0.936-1.005)	0.093
NLR	1.223 (1.111-1.346)	**0.000**	1.105 (0.963-1.268)	0.154
TNM (S_1~2_ vs S_3_)	2.364 (1.378-4.053)	**0.002**	1.726 (1.185-2.513)	**0.004**
SII (Low vs High)	2.498 (1.491-4.183)	**0.001**	1.583 (0.977-2.565)	0.062

PAR, platelet to albumin ratio; PNI, prognostic Nutritional Index; NLR, neutrophil to lymphocyte ratio; TNM S1~2, TNM stage I ~ TNM stage II ; SII, systemic immune-inflammation index SII = (P × N)/L, where P, N, and L refer to peripheral platelet, neutrophil, and lymphocyte counts, respectively.

Bold values: P value has statistical significance.

### 3.4 Relationship between SII and early recurrence

**Figure 5:**
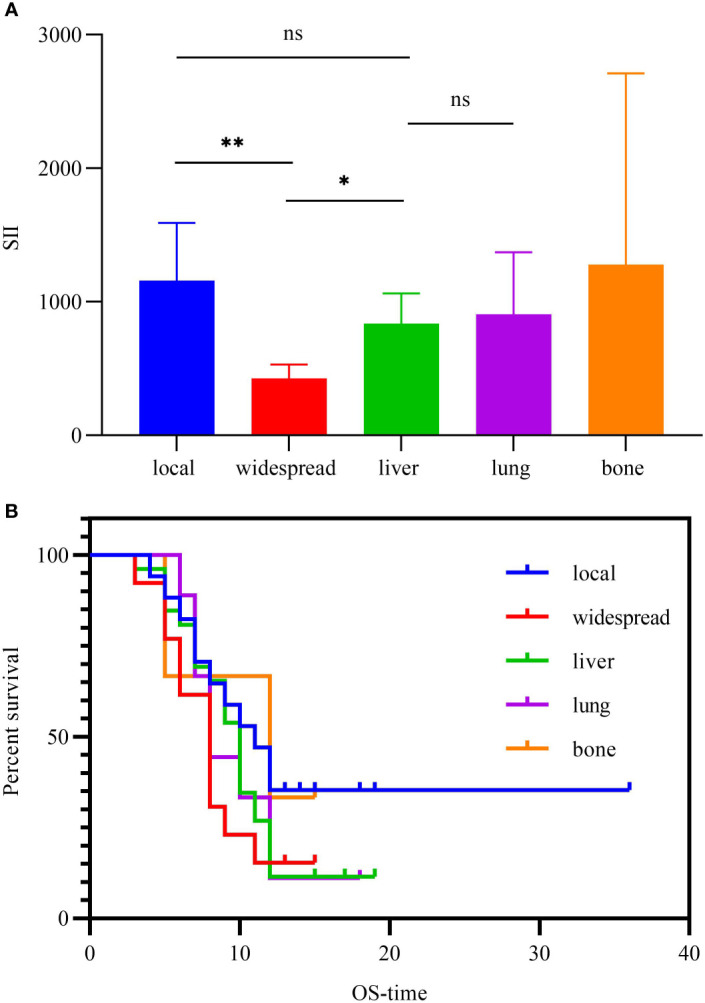
**(A)** Histogram of SII according to site of recurrence in patients who relapsed within one year. **(B)** Kaplan-Meier curves for OS according to site of recurrence in patients who relapsed within one year. *P<0.05, **P<0.01, "ns" represents no difference.

Generally, recurrence within 1 year after resection could be accepted as *de novo* recurrence. We detected 68 patients recurrences within 1 year in these patients during the period of follow-up. The SII values were compared among the different site of recurrence. 13 patients with widespread recurrences (widespread recurrences defined as occurrence of ≥2 metastatic sites) patients had lower SII values than in other groups (**Figure 5A**). To study whether recurrence locations were associated with outcome, OS were compared based on the group of local, widespread, liver, lung and bone (**Figure 5B**). Through the pairwise comparisons of different groups, we found that patients with widespread recurrences had lower SII and worse prognosis, which is contrary to the previous findings in our total cohort.

## 4 Discussion

PHC is the common type of PDAC, which is defined according to the location of the tumor, and was easy to invade the peripheral blood vessels because of the special anatomy (7). According to NCCN guidelines, surgical resection should be considered for patients with resectable and borderline resectable PDAC (2). We had accumulated a certain number of patients with conventional pancreaticoduodenectomy or PD with allogeneic venous replacement, based on the characteristics of our center. However, there is no good predictive method for the prognosis of these high-risk PHC with venous invasion, especially lack of preoperative noninvasive predictive indicators. Most patients with PHC had jaundice or cholangitis, accounting for 54% of jaundice in our cohort, which may have contributed to the low predictive efficacy of carbohydrate antigen19-9 (CA19-9) (8). Pathologic evaluation is the gold standard for predicting prognosis, but it can only be properly evaluated postoperatively. In recent years, systemic inflammatory factors and nutritional factors play a key role in cancer development, therapeutic effects and long-term survival (5, 9). We incorporated preoperative inflammatory markers and tumor markers of pancreatic cancer patients into the machine learning XGBoost model and found that SII was a factor strongly associated with OS.

According to previous studies, innate immunity plays an important role in PDAC (10). Lymphocytes, neutrophils, and platelets are all important players in innate immunity. Therefore, we aimed to investigate the differences in peripheral immunity and the prognostic significance of PHC, and the validation was compared with TNM staging system. The currently well-studied prognostic nutritional factors PNI and PAR were also included (5, 11, 12).

It was found that SII could predict the survival and recurrence of patients with PHC in our cohort. The prediction ability of SII is only inferior to that of TNM staging system. In contrast, SII is a preoperative indicator that is not affected by subsequent treatment and is better obtained. Moreover, serum inflammatory markers such as SII are readily available in clinical practice. Although SII shows good prediction of total OS and DFS, it showed different results in 1-year survival and 1-year recurrence. In patients who relapse within a year, the lower the SII, the higher the likelihood of extensive relapse and poorer survival. Only TNM stage III was an independent recurrence factor in these high-risk patients.

Neutrophils have been found to be widely associated with tumor development in recent studies (13–15). As an important player in innate immunity, neutrophils contribute to tumor progression by promoting cell proliferation, angiogenesis, tissue remodeling, immunosuppression, and metastasis (16). Neutrophils are short-lived phagocytes (17), and differentially distributed in peripheral blood and tumor tissues of PHC (14). What’s more, neutrophils were divided into many subsets by Single-cell RNA-seq analysis in recent studies. Evidence suggests that various subtypes have distinct functions (14). For patients with TNM stage III, peripheral blood neutrophil counts may not reflect the true immune status of tumor tissue.

The immune microenvironment of pancreatic cancer is extremely complex, and the immune infiltration of pancreatic cancer does not well reflect its immunological response as well (18). Pancreatic cancer is a type of tumor with extensive infiltration of immunosuppressive cells in tumour microenvironment (19). FOXP3^+^ regulatory T cells (Tregs) have been found to be associated with survival in patients with pancreatic cancer in existing studies (20). Further, high infiltration of CD3^+^CD8^−^ (mainly CD4^+^) T cells was associated with improved patient survival, whilst cytotoxic CD3^+^CD8^+^ T cell infiltration did not have an impact on overall survival (21). Lymphocytes are not effective in predicting the prognosis of pancreatic cancer and remain controversial.

In our study, we discovered that SII was able to predict both the survival and recurrence of patients with PHC, with a prediction ability that was only slightly lower than that of the TNM staging system. One of the advantages of SII over TNM staging is that it is a preoperative indicator and is not affected by subsequent treatment, making it easier to obtain. Additionally, serum inflammatory markers like SII are readily available in clinical practice. However, we did observe some differences in the predictive ability of SII in 1-year survival and recurrence. For instance, in patients who relapsed within a year, those with lower SII levels were more likely to experience extensive relapse and poorer survival outcomes. Only TNM stage III was found to be an independent recurrence factor in these high-risk patients.

Platelets also contribute to innate immunity to affect adaptive immune responses by expressing a wide range of functional immune receptors or direct influence (22).Tumor cells can induce platelet activation and aggregation (23). The interaction of tumor cells with platelets is a prerequisite for hematogenous metastatic spread (24). Emerging evidence suggests that platelets mediate tumor cell growth, angiogenesis, and proliferation (25).

Peripheral immune status is closely associated with tumor development, which has also been confirmed in previous studies. However, it is not easy to equate the peripheral immune status with the survival and recurrence of patients. The predictive ability of SII is poor in PHC with vascular invasion. As a hypovascular tumor (26), pancreatic cancer also has limited response to immune checkpoint blockade or chimeric antigen receptor T (27). Neutrophil counts, on the other hand, do not fully represent their function. Those may be the important reasons that SII could not predict tumor relapse well in high-risk patients within 1 year, especially in PHC with TNM stage III. Although including platelets, neutrophils, and lymphocytes, SII only reflects the relationship between body’s immunity and tumors to some extent. The mechanism of local inflammatory immune and tumor promotion in PHC with venous invasion is complex and needs further study.

## 5 Conclusion

In conclusion, operative therapy is the effective treatment for PHC with or without venous invasion. However, some patients may occur disease progression or recurrences after the therapies. Our study identified a high preoperative SII may be a predictor of poor prognosis and early recurrence. However, for high-risk patients with TNM SIII, low SII is not a protective factor, suggesting that more attentions are needed to figure out those patients and develop an individualized treatment plan.

## 6 Limitation

There were several limitations to our study. First, vascular replacement surgery for pancreatic cancer is rare worldwide and the sample size of the patients were not perfect. Secondly, this study is a single-center retrospective study, which may lead to missing data and selection bias. Finally, Cox regression was applied for analysis in this article, however, incomplete and biased data may also lead to less accurate results of the analysis. We will also conduct a prospective multicenter study to validate the results of this study.

## Data availability statement

The original contributions presented in the study are included in the article/supplementary material. Further inquiries can be directed to the corresponding authors.

## Ethics statement

The studies involving human participants were reviewed and approved by Ethnic Committee and Committee for Clinical Application of Medical Technology of Chaoyang Hospital (No. 2020-D-301). The patients/participants provided their written informed consent to participate in this study.

## Author contributions

(I) Conception and design: QC, SR, SC. (II)Administrative support: QH, RL. (III) Provision of study materials or patients: SC. (IV) Collection and assembly of data: JH, DW, BL. (V) Data analysis and interpretation: QC, SC, SR. (VI) Manuscript writing: All authors. (VII) All authors contributed to the article and approved the submitted version.

## References

[1] Klein AP. Pancreatic cancer epidemiology: understanding the role of lifestyle and inherited risk factors. Nat Rev Gastroenterol Hepatol (2021) 18(7):493–502. doi: 10.1038/s41575-021-00457-x 34002083 PMC9265847

[2] Tempero MA Malafa MP Al-Hawary M Behrman SW Benson AB Cardin DB. Pancreatic adenocarcinoma, version 2.2021, NCCN clinical practice guidelines in oncology. J Natl Compr Canc Netw (2021) 19(4):439–57. doi: 10.6004/jnccn.2021.0017 33845462

[3] Pancreatic Surgery Group SBoCMA Digital Medical Branch of Chinese Medical A Digital Intelligent Surgery Professional Committee of Chinese Research Hospital A Pancreatic Diseases Professional Committee of Chinese Research Hospital A [Chinese expert consensus on digital intelligent precise diagnosis and treatment of pancreatic surgical diseases (2022 edition)]. Zhonghua Wai Ke Za Zhi (2022) 60(10):881–7. doi: 10.3760/cma.j.cn112139-20220523-00234 36207975

[4] Banna GL Friedlaender A Tagliamento M Mollica V Cortellini A Rebuzzi SE. Biological rationale for peripheral blood cell-derived inflammatory indices and related prognostic scores in patients with advanced non-Small-Cell lung cancer. Curr Oncol Rep (2022) 24(12):1851–62. doi: 10.1007/s11912-022-01335-8 36255605

[5] Petzel MQB Hoffman L. Nutrition implications for long-term survivors of pancreatic cancer surgery. Nutr Clin Pract (2017) 32(5):588–98. doi: 10.1177/0884533617722929 29927530

[6] Yuan KC Tsai LW Lee KH Cheng YW Hsu SC Lo YS. The development an artificial intelligence algorithm for early sepsis diagnosis in the intensive care unit. Int J Med Inform (2020) 141:104176. doi: 10.1016/j.ijmedinf.2020.104176 32485555

[7] Henry BM Skinningsrud B Saganiak K Pekala PA Walocha JA Tomaszewski KA. Development of the human pancreas and its vasculature - an integrated review covering anatomical, embryological, histological, and molecular aspects. Ann Anat (2019) 221:115–24. doi: 10.1016/j.aanat.2018.09.008 30300687

[8] Ney A Garcia-Sampedro A Goodchild G Acedo P Fusai G Pereira SP. Biliary strictures and cholangiocarcinoma - untangling a diagnostic conundrum. Front Oncol (2021) 11:699401. doi: 10.3389/fonc.2021.699401 34660269 PMC8515053

[9] Padoan A Plebani M Basso D. Inflammation and pancreatic cancer: focus on metabolism, cytokines, and immunity. Int J Mol Sci (2019) 20(3):676. doi: 10.3390/ijms20030676 PMC6387440 30764482

[10] Hinshaw DC Shevde LA. The tumor microenvironment innately modulates cancer progression. Cancer Res (2019) 79(18):4557–66. doi: 10.1158/0008-5472.CAN-18-3962 PMC6744958 31350295

[11] Shirai Y Shiba H Haruki K Horiuchi T Saito N Fujiwara Y. Preoperative platelet-to-Albumin ratio predicts prognosis of patients with pancreatic ductal adenocarcinoma after pancreatic resection. Anticancer Res (2017) 37(2):787–93. doi: 10.21873/anticanres.11378 28179331

[12] Bullock AF Greenley SL McKenzie GAG Paton LW Johnson MJ. Relationship between markers of malnutrition and clinical outcomes in older adults with cancer: systematic review, narrative synthesis and meta-analysis. Eur J Clin Nutr (2020) 74(11):1519–35. doi: 10.1038/s41430-020-0629-0 PMC7606134 32366995

[13] Wang X Hu LP Qin WT Yang Q Chen DY Li Q. Identification of a subset of immunosuppressive P2RX1-negative neutrophils in pancreatic cancer liver metastasis. Nat Commun (2021) 12(1):174. doi: 10.1038/s41467-020-20447-y 33420030 PMC7794439

[14] Wang L Liu Y Dai Y Tang X Yin T Wang C. Single-cell RNA-seq analysis reveals BHLHE40-driven pro-tumour neutrophils with hyperactivated glycolysis in pancreatic tumour microenvironment. Gut (2022) 72(5):958–71. doi: 10.1136/gutjnl-2021-326070 PMC10086491 35688610

[15] Cerezo-Wallis D Hidalgo A. A hypoxic ride for neutrophils in PDAC. Gut (2022) 72(5):817–8. doi: 10.1136/gutjnl-2022-327953 35817554

[16] Jaillon S Ponzetta A Di Mitri D Santoni A Bonecchi R Mantovani A. Neutrophil diversity and plasticity in tumour progression and therapy. Nat Rev Cancer (2020) 20(9):485–503. doi: 10.1038/s41568-020-0281-y 32694624

[17] Hidalgo A Chilvers ER Summers C Koenderman L. The neutrophil life cycle. Trends Immunol (2019) 40(7):584–97. doi: 10.1016/j.it.2019.04.013 31153737

[18] Deng ZL Zhou DZ Cao SJ Li Q Zhang JF Xie H. Development and validation of an inflammatory response-related gene signature for predicting the prognosis of pancreatic adenocarcinoma. Inflammation (2022) 45(4):1732–51. doi: 10.1007/s10753-022-01657-6 35322324

[19] Looi CK Chung FF Leong CO Wong SF Rosli R Mai CW. Therapeutic challenges and current immunomodulatory strategies in targeting the immunosuppressive pancreatic tumor microenvironment. J Exp Clin Cancer Res (2019) 38(1):162. doi: 10.1186/s13046-019-1153-8 30987642 PMC6463646

[20] Chellappa S Hugenschmidt H Hagness M Line PD Labori KJ Wiedswang G. Regulatory T cells that co-express RORgammat and FOXP3 are pro-inflammatory and immunosuppressive and expand in human pancreatic cancer. Oncoimmunology (2016) 5(4):e1102828. doi: 10.1080/2162402X.2015.1102828 27141387 PMC4839385

[21] Brouwer T Ijsselsteijn M Oosting J Ruano D van der Ploeg M Dijk F. A paradoxical role for regulatory T cells in the tumor microenvironment of pancreatic cancer. Cancers (Basel) (2022) 14(16):3862. doi: 10.3390/cancers14163862 PMC9405872 36010856

[22] Maouia A Rebetz J Kapur R Semple JW. The immune nature of platelets revisited. Transfus Med Rev (2020) 34(4):209–20. doi: 10.1016/j.tmrv.2020.09.005 PMC7501063 33051111

[23] Schlesinger M. Role of platelets and platelet receptors in cancer metastasis. J Hematol Oncol (2018) 11(1):125. doi: 10.1186/s13045-018-0669-2 30305116 PMC6180572

[24] Haemmerle M Stone RL Menter DG Afshar-Kharghan V Sood AK. The platelet lifeline to cancer: challenges and opportunities. Cancer Cell (2018) 33(6):965–83. doi: 10.1016/j.ccell.2018.03.002 PMC5997503 29657130

[25] Mitrugno A Tassi Yunga S Sylman JL Zilberman-Rudenko J Shirai T Hebert JF. The role of coagulation and platelets in colon cancer-associated thrombosis. Am J Physiol Cell Physiol (2019) 316(2):C264–73. doi: 10.1152/ajpcell.00367.2018 PMC6397342 30462538

[26] Nguyen DT Lee E Alimperti S Norgard RJ Wong A Lee JJ. A biomimetic pancreatic cancer on-chip reveals endothelial ablation *via* ALK7 signaling. Sci Adv (2019) 5(8):eaav6789. doi: 10.1126/sciadv.aav6789 31489365 PMC6713506

[27] Schizas D Charalampakis N Kole C Economopoulou P Koustas E Gkotsis E. Immunotherapy for pancreatic cancer: a 2020 update. Cancer Treat Rev (2020) 86:102016. doi: 10.1016/j.ctrv.2020.102016 32247999

